# Humoral immune response to Shiga Toxin 2 (Stx2) among Brazilian urban children with hemolytic uremic syndrome and healthy controls

**DOI:** 10.1186/1471-2334-14-320

**Published:** 2014-06-11

**Authors:** Mirian Guirro, Roxane Maria Fontes Piazza, Renato Lopes de Souza, Beatriz Ernestina Cabilio Guth

**Affiliations:** 1 Department of Microbiology, Immunology, Parasitology, Escola Paulista de Medicina, Universidade Federal de São Paulo, São Paulo, Brazil; 2 Bacteriology Laboratory, Instituto Butantan, São Paulo, Brazil; 3 Pediatric Intensive Care Unit, Department of Pediatrics, Escola Paulista de Medicina, Universidade Federal de São Paulo, São Paulo, Brazil

## Abstract

**Background:**

Shiga toxin (Stx)-producing *Escherichia coli* (STEC) infection is associated with hemolytic uremic syndrome (HUS), the main cause of acute renal failure in early childhood. Stx is essential in the pathogenesis of HUS, which has been mostly related to Stx2-producing isolates. Very limited data exist on the immune response to STEC in the Brazilian population. In this study, the prevalence of immunoglobulin G (IgG) antibodies to Stx2 was investigated in sera of children diagnosed with HUS and of healthy children in the city of São Paulo, Brazil.

**Methods:**

IgG-antibody reactivity to Stx2 was determined by immunoblotting (WB) and enzyme-linked immunosorbent assay (ELISA) in sera from 13 children with HUS aged 8 months to 6 years and 54 healthy urban children aged 5 months to 7 years.

**Results:**

A positive immune response to the A and B subunits of Stx2 was observed in 46.1% HUS patients and in 16.6% healthy individuals by WB. All HUS patients and 62.9% healthy children showed IgG antibodies to the Stx2 A subunit. The frequency of antibodies to both subunits or only to the A subunit of Stx2 was significantly higher in HUS patients than controls (p < 0.05). Also, the mean OD value obtained by ELISA was higher in that group. Considering children’s age, the frequency of reactivity to either the A subunit or both subunits of Stx2 was considerably higher in HUS children up to three years old compared to controls in the same age range. Moreover, in almost 37% of healthy children, no immune response to Stx2 was detected independently of the child’s age.

**Conclusions:**

The seroepidemiolgy of anti-Stx2 antibodies was described for the first time in healthy children and children with HUS in Brazil. The percentage of individuals showing antibodies against Stx2 was higher among HUS patients than controls, and in spite of the low number of notified HUS cases, STEC strains are circulating in our settings. In addition, the results obtained also corroborated previous data on the increased sensitivity and specificity of WB compared to toxin-based enzyme immunoassays.

## Background

Shiga toxin-producing *Escherichia coli* (STEC) infection can induce hemolytic uremic syndrome (HUS), a thrombotic microangiopathy characterized by acute renal failure, thrombocytopenia and hemolytic anemia. O157:H7 is the most prominent STEC serotype implicated in serious outbreaks and sporadic cases of HUS. However, in the last decade, a wide range of non-O157 STEC serotypes have shown a significant etiological role in the illness [1,2]. Worldwide, there is substantial geographic variation in the prevalence of STEC serotypes as well as in the incidence of HUS. In Brazil, human STEC infections have been linked mostly to sporadic cases of non-bloody diarrhea associated mainly with non-O157 strains [3,4]. However, HUS cases associated with O157 as well as non-O157 STEC infections have more recently been described in São Paulo State [5-8]. The most important virulence property of STEC is its ability to produce Shiga toxins (Stx), central in the pathogenesis of HUS [9]. Stx consist of one enzymatically active A subunit (32 kDa) linked to a pentamer of B subunits (7.5 kDa), and are produced during mucosal colonization and delivered to the circulation [10]. There is increasing evidence demonstrating the damage caused to vascular endothelial cells in various organs and tissues, including kidneys and gastrointestinal tract [11,12]. The toxin family contains two major groups that are serologically distinct, called Stx1 and Stx2. The latter has multiple subtypes or variants in a range of combinations [13]. Among the Stx produced by human STEC isolates, Stx2 and Stx2c show the highest association with severe cases of HUS [14-16].

Protective immunity to STEC infection is likely to result from the interplay between antibodies that inhibit colonization of the bowel and those that neutralize Stx [17]. Experimental and clinical findings suggest that Stx antibodies can develop a role in the protective immune response as well as contributing to HUS resistance [17-19]. However, several epidemiological analyses have demonstrated that approximately 75% of HUS cases occur in children less than 5 years old, suggesting that the disease may be associated with the absence of preexisting immunity in the pediatric population. Neutralization assay in cell cultures was the earliest approach for detecting antibodies to Stx in human serum. However, some studies found nonspecific neutralizing activity in this assay due to a lipoprotein component of the serum [20]. To overcome these limitations, other assays such as enzyme-linked immunosorbent assay (ELISA) and Western blot (WB) have been employed [17,20-22].

Information about the nature and measurement of the immune response directed against Stx in Brazil is limited. Palmeira et al. [23] investigated the ability of sera obtained from healthy adults to neutralize Stx2. Thus, seroepidemiological data regarding anti-Stx2 antibodies in the Brazilian population and the use of serodiagnosis could help to explain the epidemiological profile of the illness. Therefore, the aim of the present study was to investigate the prevalence of IgG antibodies to Stx2 in sera of children diagnosed with HUS and of healthy children (control population) in the city of São Paulo, SP, Brazil, using the accurate and sensitive techniques WB and ELISA, outlining the first report on the analysis of Stx antibodies in children in Brazil.

## Methods

### Human serum samples – patients and controls

Sera were obtained from 13 patients aged 8 months to 6 years, presenting with typical symptoms of HUS. All patients developed HUS after gastroenteritis, with diarrheal prodromes for a median of 6.4 days [8]. The samples were collected in pediatric intensive care units in the city of São Paulo during 2001 and 2005. Serum was collected at admission, in the acute phase of the disease. The patients were not associated with a STEC outbreak. Serum samples were collected from 54 urban healthy children aged 5 months to 7 years with no signs of infectious disease or symptoms of gastrointestinal disorders, at least for the preceding 30 days, who were seen at the Hospital São Paulo outpatient clinic [8]. All serum samples were stored at -20°C until use. The study was approved by the Research Ethics Committee at the Universidade Federal de São Paulo (UNIFESP), and parents gave informed consent for their children to be included in the study.

### Stx2

The purified protein was purchased from Tufts New England Medical Center (Tufts University School of Medicine) [24].

### Immunoblotting (WB)

This assay was adapted from the method reported by Karmali et al. [17]. Briefly, a standard concentration of purified Stx2 (3 μg/10 μL) was resolved into its A and B subunits by SDS-PAGE. Proteins were transferred to nitrocellulose membranes, which were then cut into longitudinal strips, blocked with 0.01 M phosphate buffered saline (PBS), pH 7.2, containing 5% skim milk and 2% bovine serum albumin (BSA) (blocking buffer), and incubated for 2 h with each serum sample diluted 1:250 in blocking buffer. After washes in PBS-0.05% Tween-20, the strips were incubated with a 1:2000 dilution of peroxidase-conjugated anti-human IgG (Sigma, St Louis, MO, USA) in blocking buffer. A polyclonal antibody obtained from a rabbit immunized with Stx2 [25] was used as the positive control for the assay, and this membrane was incubated with a 1:2000 dilution of peroxidase-conjugated anti-rabbit IgG (Sigma, St Louis, MO, USA) in blocking buffer. Antigen-antibody complexes were visualized with the enhanced chemiluminiscent detection system (ECL, Amersham Life Science), and positive and negative responses were qualitatively analyzed. Each serum sample was assayed at least twice.

### ELISA

The assay was based on the method described by Karmali et al. [19], and prior to its application, a dose–response curve determined the amount of Stx2 to be used as antigen. Microtiter plates were coated by overnight incubation at 4°C with 100 μL of 1 μg/mL Stx2 in PBS. Nonspecific reactions were blocked by incubation with PBS containing 2% BSA at 37°C for 2 h. The wells were washed and incubated with serum samples diluted 1:500 in PBS-BSA at 37°C for 2 h. After washes, the plates were incubated with 1:2000 peroxidase-conjugated anti-human IgG (Sigma, St Louis, MO, USA) diluted in PBS-BSA at 37°C for 2 h. Next, 100 μL of substrate solution (0.1 M citrate-phosphate, pH 5; 1 mg/mL ο-phenylenediamine dihydrochloride - Sigma-Aldrich; 30% H_2_O_2_) were added to each well and the plates incubated for 20 min at room temperature in the dark. The reaction was stopped by the addition of 50 μL of 4 N sulfuric acid, and optical density was measured at 492 nm (OD492) using an ELISA microplate reader. Each serum sample was tested in duplicate at least in two independent experiments and the mean OD was determined.

### Statistical analysis

The significance of differences between the two groups of children obtained with WB was ascertained by Fisher’s exact test. Differences were considered to be significant when p < 0.05. The receiver operating characteristic (ROC) curve was employed for determining the cut-off value using the data obtained by ELISA, considering the highest sensitivity and specificity.

## Results

**Figure 1:**
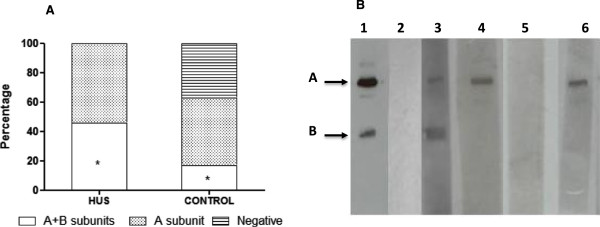
**Reactivity profile against Stx2 by Immunoblotting (WB) in HUS patients and healthy children (controls). A)** Each bar represents samples reactive to A and B subunits or only A subunit or non-reactive. * Significant differences were observed between the groups (p = 0.032). **B)** Representative immunoblotting strips from HUS patients (lanes 3 and 4) and control sera (lanes 5 and 6) reactive to Stx2. Immunoblotting reaction was carried out by incubating nitrocellulose membranes, containing purified Stx2 toxin subjected to 12.5% SDS-PAGE, with sera from HUS patients and healthy individuals followed by incubation with goat anti-human IgG. Lanes 1 and 2 represent positive and negative controls of the assay, respectively, purified toxin incubated with rabbit anti-Stx2 polyclonal antibody (lane 1) or pre-immune rabbit serum (lane 2), followed by peroxidase-conjugate anti-rabbit IgG. The arrows represent the A subunit (32 kDa) and B subunit (7.5 kDa) of Stx2.

**Figure 2:**
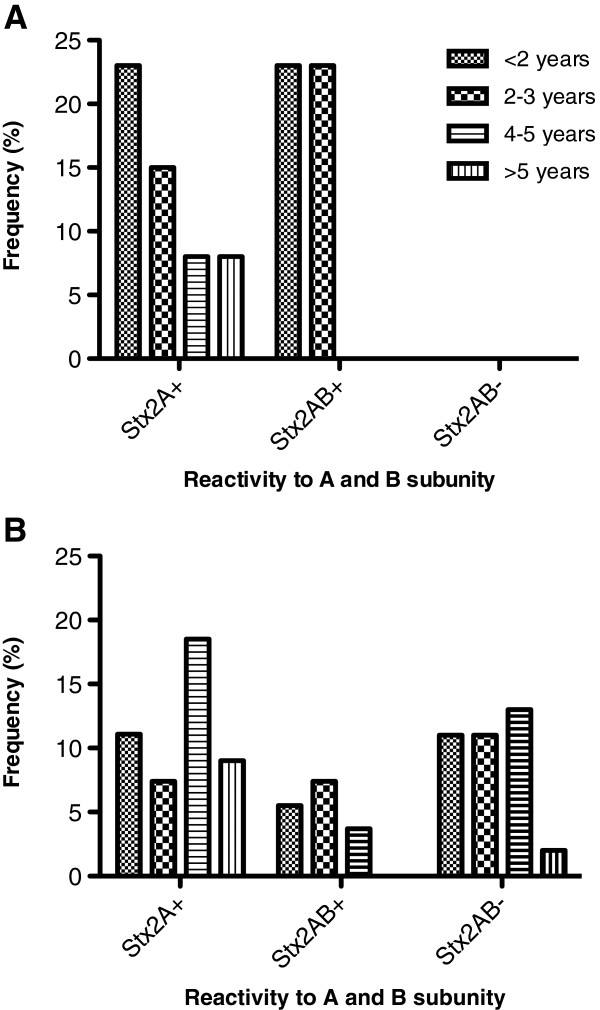
Frequency of serum reactivity to A and B subunits of Stx2 according to the age of HUS patients (A) and healthy children (controls) (B).

**Figure 3:**
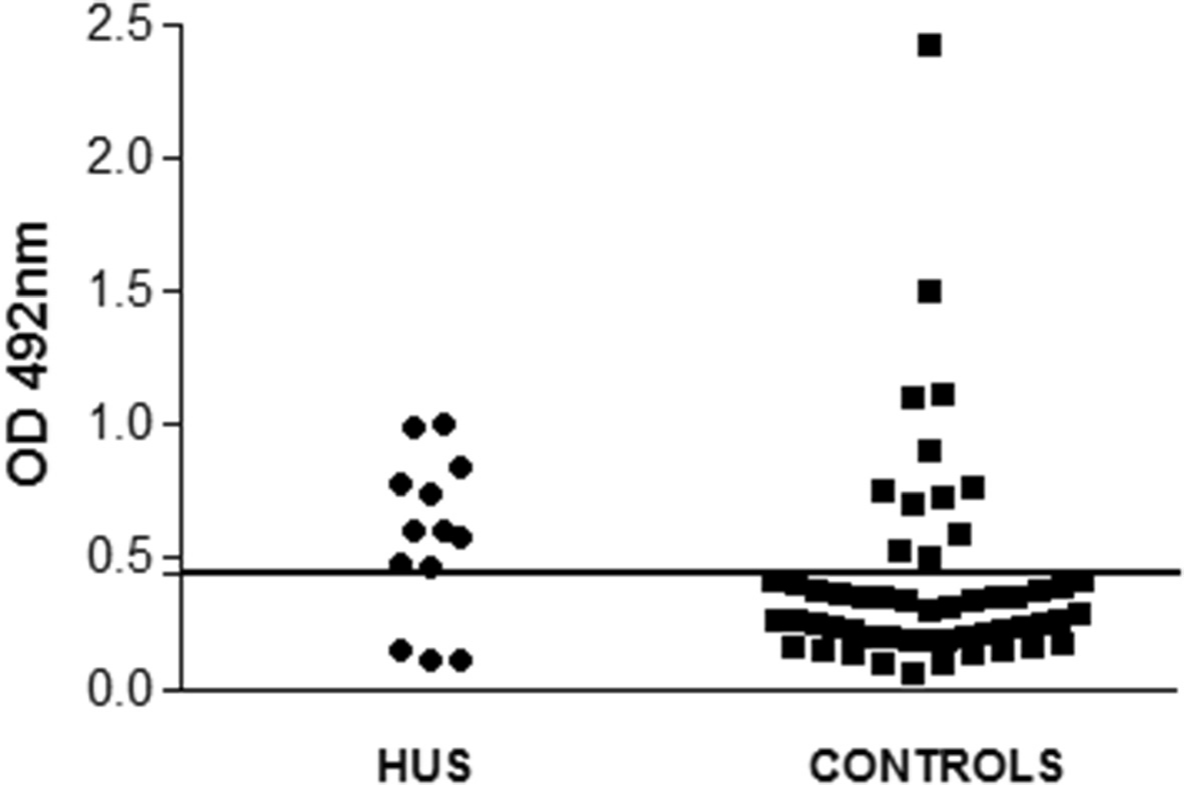
**Serum reactivity to Stx2 assessed by ELISA and optical densities determined at 492 nm.** The optical density values representing the level of IgG against Stx2 are individually shown and correspond to the mean OD of two independent assays performed in duplicate wells. The horizontal line (OD = 0.443) represents the cut-off value.

The IgG reactivity profile was first analyzed by WB, and a positive immune response to the A and B subunits of Stx2 was observed in six (46.1%) HUS patients and nine (16.6%) healthy individuals (Figure 1A). All 13 HUS patients and 34 (62.9%) healthy children showed IgG antibodies to the Stx2 A subunit. Considering the response to both subunits or only the A subunit, the frequency of antibodies was significantly higher in HUS patients than controls (p = 0.032 and p = 0.0055, respectively). Antibodies reacting only with the B subunit were not detected in sera of either HUS patients or healthy children. A representative IgG response to Stx2 detected by WB in sera of HUS patients and control subjects is shown in Figure 1B. It is worth mentioning that considering the child’s age, the frequency of reactivity either to A subunit or both subunits of Stx2 was considerably higher in HUS children less than 2 and 2–3 years old compared to controls in the same age range (Figure 2A and B). Antibodies to A subunit were found in sera of healthy children of all ages, but the frequency of response was higher in older children. Moreover, it is interesting to note that in almost 37% of healthy children, no immune response to Stx2 was detected independently of the child’s age.The serum levels of IgG antibodies reactive to Stx2 as determined by ELISA are shown in Figure 3, and a wide range of OD values was observed. The cut-off value was calculated using the ROC curve, by means of 0.769 for sensitivity and 0.778 for specificity. Using these criteria, 10 (76.9%) HUS children and 12 (22.2%) healthy individuals had OD values above the cut-off (p = 0.00039). In addition, the mean OD value was higher in the HUS group than the controls (0.56 and 0.40, respectively).

Ten HUS patients identified as showing antibodies to A or AB subunits by WB were also positive by ELISA (Table 1). In controls, 11 (20.3%) children were reactive to Stx2 by WB and were also positive by ELISA, and 19 (35.18%) children did not show any response to Stx2 either by WB or ELISA. On the other hand, 23 (42.6%) healthy children identified as showing IgG to Stx2 by WB were negative by ELISA, and all of them were only reactive to the Stx2 A subunit except for one child. Therefore, relative sensitivity and specificity of WB compared to ELISA were 100 versus 83% and 81 versus 83%, respectively.

**Table 1:** Relationship between results of antibody response to A and B subunits of Stx2 by Western blot (WB) and ELISA (E)

**WB**^**a**^	**No. of sera from HUS**		**No. of sera from controls**	
	**E**^**+b**^	**E**^**-c**^	**E**^**+**^	**E**^**-**^
A^+^ / AB^+^	10	3	11	23
AB^-^	0	0	1	19

^a^A^+^/AB^+^, Western blot positive by any parameter whether AB positive or only A positive; AB^-^, Western blot negative.

^b^E^+^, ELISA positive.

^c^E^-^, ELISA negative.

## Discussion

STEC infections and HUS are the main cause of acute kidney disease in children under five in some Latin America countries such as Argentina [16]. In spite of Brazil being next to Argentina, a low incidence of HUS cases has been identified so far in Brazil, but the importance of O157 as an agent of severe disease has also been highlighted [8]. Several reasons can impact the epidemiological profile of STEC infections and HUS, including climatic conditions, exposure to animal sources of infection or food manufacturing and preparation processes [26]. Moreover, it has been previously proposed that existing anti-Stx antibodies decrease the risk of HUS. The concept that these antibodies develop a role in protective immunity comes from experimental and clinical findings [17]. Some studies have detected an increasing frequency of antibodies to Stx in the higher age population, which is in general refractory to HUS [17,18]. In addition, the immunization of laboratory animals with the Stx B subunit and fusion proteins induced neutralizing antibodies that protected mice against a challenge of *E. coli* O157:H7 or toxins [27,28].

Although several hypotheses have been proposed to try to explain the low incidence of HUS cases in Brazil, no studies have been previously performed on children’s immune response to Stx in our settings. Moreover, seroepidemiological studies of the immune response to Stx can be useful for indirectly assessing the degree of exposure to STEC in different populations in different geographic locations [17]. By using a sensitive assay, WB, we showed that a high frequency of IgG antibodies directed to Stx2 was present in the sera of HUS patients and in 62.9% of healthy children. Fernández-Brando et al. [22] detected 67% reactivity to Stx2 by WB in sera from healthy children in Argentina, where STEC/Stx2 is highly endemic [16]. In Germany, Ludwig et al. [21] detected an anti-Stx2 response in 71% of patients infected by a Stx2-producing strain and in 10% of control sera. Karmali et al. [17] found anti-Stx2 antibodies in 93% of HUS patients and 46% of urban residents in Canada. In Germany and Canada, STEC has been implicated in large outbreaks as well as in sporadic cases of hemorrhagic colitis and HUS [29-31]. The observed frequency of antibodies directed to Stx2 can reflect the prevalence of *stx*_2_ genes in STEC strains endemic in a particular area.

Despite the high frequency of IgG antibodies directed to Stx2 identified in the present study, we observed that the percentage of individuals showing antibodies against Stx2 was higher among HUS patients than controls (p < 0.05). Also, the mean OD value obtained by ELISA was higher in that group. Considering that elevated antibody levels can indicate an active or recent infection [32], these results suggest that the STEC infection causing HUS elicited the immune response.

Stx-producing strains were isolated from only three HUS patients presently studied [8]. Nevertheless, sera from patients, from whom Stx2-producing O157 and O165 strains were isolated, showed antibodies to A and B subunits, and serum of the other patient, from whom an O26 Stx1-producing strain was recovered, reacted only with the A subunit of Stx.

It was interesting to observe that ten of 13 HUS patients and 11 of 34 children reactive to Stx2 by WB were found to be positive by ELISA, showing a stronger association between the response detected by WB and ELISA in the HUS group compared to healthy children. These results are similar to those described by others and may be due to the higher levels of antibodies in patients’ sera [22].

The predominance of reactivity to A or B subunit is variable according to different studies on immune response to Stx2. In the present study, HUS patients and control individuals reacted predominantly with the A subunit of Stx2, but a positive immune response to A and B subunits was observed in 46.1% of HUS patients and 16.6% of controls (p < 0.05), while antibodies reacting only to the B subunit were not detected in sera of either HUS patients or healthy children. Ludwig et al. [21] found that 85% of HUS patient serum samples positive for anti-Stx2 IgG were reactive with the A subunit and 15% recognized the B subunit; in the control group, 45% of serum samples reacted with the A subunit and 55% recognized the B subunit. Karmali et al. [17] showed that 70% of serum samples obtained from urban residents in Canada were reactive to the AB subunits, 7.5% only to the A subunit and 22.2% only to the B subunit. Among HUS patients in Canada, all samples reacted with both the A and B subunits. Fernández-Brando et al. [22] demonstrated that 70% of HUS patients and 47% of controls in Argentina reacted to the A subunit, and a response to the B subunit was detected in 72% of HUS patients and 53% of controls. In addition, they also found that antibody response to the B subunit tended to decrease faster than the antibody response to the A subunit. The differences in response to Stx2 A and B subunits observed in the studies described so far might be related to several factors, including differences in exposure, age of patients and even methodological approaches.

Almost half of the HUS patients (46%) studied herein were less than two years of age [8], confirming the higher incidence of HUS in the youngest population. It is interesting that among the 23 control children up to two years old, eight showed antibodies to the A subunit, seven were also positive with regard to the B subunit of Stx2, and no response was detected among the others. One may suggest that these antibodies might have been transferred from their mother across the placenta or by breast milk [33]. However, the data obtained in this study cannot support the notion that these transferred Stx2 antibodies afford protection to children. A relatively high frequency of serum samples of healthy adults living in São Paulo City showed neutralizing activity against Stx2, as demonstrated by Vero cell culture assays [23]. We tested the serum of two HUS patients and two controls, but did not detect neutralizing activity in HUS serum, although a partial neutralization was observed in one control serum (data not shown). Scotland et al. [34] also detected neutralizing activity in control serum against Stx2. However, this response was attributed to the nonspecific activity of a high-density lipoprotein present in human serum rather than to specific antibodies [20].

## Conclusion

The data presented herein describe for the first time the seroepidemiolgy of anti-Stx antibodies in healthy children and children with HUS in Brazil, making an important contribution to understanding the epidemiology of STEC infections. The percentage of individuals showing antibodies against Stx2 was higher among HUS patients than controls, and in spite of the small number of reported HUS cases, STEC strains are circulating in our settings. In addition, the results obtained also corroborated the previous data on the increased sensitivity and specificity of WB compared to toxin-based enzyme immunoassays.

## Competing interests

The authors declare that they have no competing interests.

## Authors’ contributions

Laboratory investigations and data analysis were performed by MG. RMFP assisted in the development of ELISA assays and preparation of manuscript. RLS was responsible for collection of sera and clinical information. BECG designed the study, assisted in the development of the research proposal and preparation of manuscript. All authors read and approved the final manuscript.

## Pre-publication history

The pre-publication history for this paper can be accessed here:

http://www.biomedcentral.com/1471-2334/14/320/prepub
